# Functional Materials Made by Combining Hydrogels (Cross-Linked Polyacrylamides) and Conducting Polymers (Polyanilines)—A Critical Review

**DOI:** 10.3390/polym15102240

**Published:** 2023-05-09

**Authors:** Cesar A. Barbero

**Affiliations:** Research Institute for Energy Technologies and Advanced Materials (IITEMA), National University of Río Cuarto (UNRC)—National Council of Scientific and Technical Research (CONICET), Río Cuarto 5800, Argentina; cbarbero@exa.unrc.edu.ar; Tel.: +54-3584028768

**Keywords:** polyanilines, polyacrylamides, hydrogels, nanocomposite, semi-interpenetrated network, photothermal, sensor, actuator, supercapacitor, conductivity

## Abstract

Hydrogels made of cross-linked polyacrlyamides (cPAM) and conducting materials made of polyanilines (PANIs) are both the most widely used materials in each category. This is due to their accessible monomers, easy synthesis and excellent properties. Therefore, the combination of these materials produces composites which show enhanced properties and also synergy between the cPAM properties (e.g., elasticity) and those of PANIs (e.g., conductivity). The most common way to produce the composites is to form the gel by radical polymerization (usually by redox initiators) then incorporate the PANIs into the network by oxidative polymerization of anilines. It is often claimed that the product is a semi-interpenetrated network (s-IPN) made of linear PANIs penetrating the cPAM network. However, there is evidence that the nanopores of the hydrogel become filled with PANIs nanoparticles, producing a composite. On the other hand, swelling the cPAM in true solutions of PANIs macromolecules renders s-IPN with different properties. Technological applications of the composites have been developed, such as photothermal (PTA)/electromechanical actuators, supercapacitors, movement/pressure sensors, etc. PTA devices rely on the absorption of electromagnetic radiation (light, microwaves, radiofrequency) by PANIs, which heats up the composite, triggering the phase transition of a thermosensitive cPAM. Therefore, the synergy of properties of both polymers is beneficial.

## 1. Introduction

Cross-linked polyacrylamides (cPAM) are the materials most widely used to produce synthetic hydrogels [1], due to their simple aqueous synthesis, chemical stability and reproducible formation of hydrogels. The use of polyacrylamide as gel media for electrophoresis makes it the most commonly in situ synthesized material [2]. Moreover, using functionalized acrylamides as monomers, it is possible to produce hydrophilic [3], or hydrophobic gels [4], thermosensitive (“smart”) materials [5], polyelectrolytes bearing positive [6] or negative charges [7], etc. The hydrophilic gels swell strongly (>20.000%) in water [8], and the more hydrophobic polymers also swell in nonaqueous solvents [9]. Similar to other synthetic hydrogels, their large water retention capacity makes them useful in drug release [10], water absorption [11] and decontamination [12], sensors [13], actuators [14], etc. The gels are produced by radical polymerization (homo or copolymerization) of the monomer in the presence of a crosslinker (a diacrylamide or hydrophilic diacrylate) [15]. The polymerization in a concentrated (>0.1 M) solution produces a macroscopic, highly hydrated solid (hydrogel) in the shape of the container [16]. These hydrogels have meso and micropores <50 nm). Using cryogelation, macroporous hydrogels can be produced [17]. If the polymerization occurs inside micrometric-sized water domains of a water-in-oil emulsion, microspheres are produced [18]. Moreover, polymerization in dilute solution is controlled by nucleation and growth processes, producing hydrogel nanoparticles (nanogels) [19]. Growing the hydrogel from monomers grafted on solids produces thin films [20]. The acrylamides can also be photopolymerized, using photoinitiators [21], allowing the production of small shapes such as electrospun nanofibers [22] or 3D printed shapes [23,24]. As can be seen, a variety of gel shapes/sizes can be produced with the same polymerization chemistry. In addition to the ability to incorporate large quantities of water inside the hydrogel matrix, an important property is the large deswelling that occurs when some hydrogels (e.g., cPNIPAM) are heated at temperatures above a threshold (lower critical solution temperature, LCST). A linear PNIPAM polymer is soluble below the LCST and insoluble above it. In PNIPAM polymers, the change in solubility is related to the thermal rotation of the hydrophobic isopropyl group which, at the LCST, overcomes the strength of the water’s hydrogen bond with the amide group. The effect involves a hydrophobic/hydrophilic equilibrium, which is also affected by the neighboring groups. The presence of hydrophilic groups increases the LCST, while the presence of hydrophobic groups decreases it. 

Polyanilines are a family of conducting polymers which are widely used due to their ease of synthesis [25], chemical stability [26] and pH-dependent conductivity [27]. By incorporation of functional groups in the ring [28] or the amine nitrogen [29], it is possible to change the redox and pH properties, albeit always reducing the electronic conductivity. Although some functionalized anilines that have electron donating groups attached to the ring can be polymerized (e.g., o-anisidine) [30], those bearing electron withdrawing groups cannot be homopolymerized. Therefore, such monomers have to be copolymerized with aniline [31], or the group has to be attached by post-functionalization [32]. Polyaniline is hydrophilic but insoluble in water. It is only soluble in acids (e.g., formic) or in strong hydrogen bonding acceptors (e.g., N-methylpyrrolidone). On the other hand, attaching charged groups to the ring (e.g., sulfonated polyaniline [33]) makes the polymer soluble in aqueous solution. Polyanilines show high conductivity (up to 300 S/cm [34]), show two redox processes and protonation/deprotonation equilibrium. Additionally, they show optical absorption bands in the UV and visible–NIR parts of the spectrum [35]. The intrinsic electronic conductivity of PANI allows it to be heated using microwaves (e.g., 2.4 GHz) [36], radiofrequency (e.g., 30 kHz) [37], or AC/DC current [38]. The electronic and physicochemical properties of polyanilines have been applied in a myriad of technological devices [39]. The combination of cross-linked polyacrylamides and polyanilines produces functional materials with various shapes, electronic and ionic conductivities that can suffer oxidation/reduction and can be heated by irradiation with light in the UV–vis–NIR range (200–1500 nm).

Studies of conducting hydrogels produced by combining different cross-linked materials and conducting polymers, specifically polyaniline [40], have been reviewed in a general way [41,42], or focusing on one application field [43,44]. A range of conducting hydrogels can be made with different combinations of conducting polymers (PANI, PPy, PEDOT, PTh, etc.) and different hydrogel matrixes (PAA, polyacrylates, PVA, biopolymers, etc.), so the whole field is quite large. Moreover, different cPAM or PANIs materials share common physicochemical properties among them, which allows for meaningful discussion of the observed effects. On the other hand, a review dealing only with conducting hydrogels would be not comprehensive but merely descriptive, as seems to be the case with many of the reviews already published [41,42,43,44].

Therefore, the present review deals comprehensively and critically with studies on the synthesis, characterization and technological applications of conductive hydrogels which are produced by a combination of cross-linked polyacrylamides (cPAM), including copolymers with other monomers, and different polyanilines (PANIs). In this way, a discussion comparing both the physicochemical properties linked to the hydrogel matrix and those of the dispersed conducting polymer is possible. 

The search for publications was performed in Scopus^®^ and Google Scholar^®^ including only peer-reviewed articles (no conference proceedings or books were included). The search terms used were: “hydrogel and polyanilline (as polyanillines, PANI or aniline)”. This search returned >600 publications. From these, only studies related to polyacrylamides (including copolymers and substituted acrylamide monomer units) were selected. Any manuscript published before February 2023 which met the criteria is discussed.

## 2. Combination of Polyacrylamides and Polyanilines

There are three ways to combine cPAM and PANIs. One is the formation of nanocomposites which contain nanoparticles of PANIs (first phase) dispersed in a cPAM matrix (second phase). Another is the blending PANIs macromolecules with the large cPAM networks. Since PANIs are formed as linear chains which are mixed with a cross-linked network, these materials are called semi-interpenetrated networks (s-IPN). Finally, PANIs chains can be grafted onto cPAM. 

### 2.1. Synthesis 

#### 2.1.1. Nanocomposites

In situ polymerization of anilines inside cPAM (ISP) is very simple to implement and is the most extensively used method to combine PANIs and cPAM [45,46,47,48]. The hydrogel is formed by radical polymerization of an acrylamide (or acrylic acid) in the presence of a crosslinker (a compound bearing two vinyl groups in the same molecule). The most common system initiator method is redox initiation with an oxidant (e.g., APS [49]) and usually an activator (e.g., TEMED [50]). In this way, a nanoporous gel is produced. On the other hand, superporous hydrogels of cPAM can be made by cryogelation [51]. Moreover, hydrogels as thin films [52], fibers [53] or particles [54] can be produced and then combined with PANIs.

**Scheme 1:**
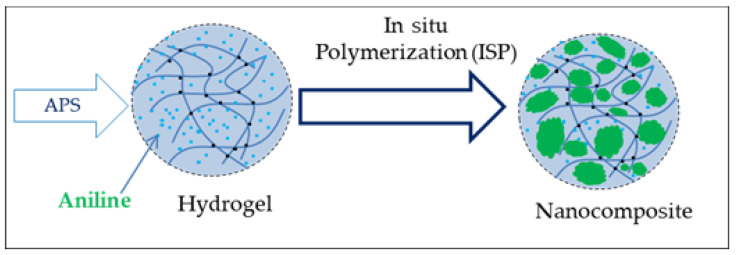
Formation of a nanocomposite by in situ polymerization (ISP) of aniline inside a porous hydrogel.

Aniline is then absorbed inside the preformed hydrogel, and the swollen gel is brought into contact with the oxidant (usually APS) in acid solution (Scheme 1).

While the reaction resembles the polymerization of aniline in solution [55], some conditions are different. The stoichiometric ratio of APS to aniline is 1.25. One mole of APS absorbs two moles of electrons from aniline, producing PANI. Moreover, 0.5 moles of electrons are additionally required to oxidize the PANI to its conductive state (emeraldine). On the other hand, a ratio of APS/aniline in excess of 1.25 could degrade the PANI chains by overoxidation [56]. Therefore, an excess of aniline is optimum. Since the diffusion of reactants inside the gel is slow, it is better to add APS to a gel loaded with aniline than vice versa. Another factor is the acidity of the reaction medium. It is possible to produce PANI in an unbuffered neutral solution since the polymerization produces protons and the pH becomes acid over time [57]. However, as with production in solution, the most common condition involves using acidic APS [47,48]. On the other hand, since APS can act as initiator of radical polymerization of acrylamides and as oxidant for aniline oxidative polymerization, a one-pot reaction of vinylic monomers, aniline and APS has been used to produce combined materials [58,59]. In this case no crosslinker is used, but a gel is obtained. Generating the gel could be achieved by grafting of PANI onto the polyacrylamide chains to produce a hybrid network and/or strong interactions through non-covalent bonds between the flexible polyacrylamide chains and rigid PANI chains. The gel can be produced by a simple radical polymerization or by a complex biomimetic self-assembly [60], with similar results. 

It is usually assumed that in situ polymerization of anilines inside a cPAM network produces s-IPN [45,46,47,48,61], but it is more likely to result in a nanocomposite of PANI NPs dispersed in the hydrogel [62,63]. Since polyanilines are insoluble in aqueous solution, the growing chains precipitate during polymerization in solution, forming nano and microparticles which aggregate into solids. When PANIs grow inside the pores of cPAM, they precipitate, thereby filling the pores [64]. SEM micrographs of the material surface show conductive (PANI) and isolating domains (cPAM). Moreover, the physicochemical properties of the polymer gel are not affected by the presence of PANI, suggesting that two phases are present. Celik and Ekici polymerized aniline inside a cPAAm cryogel [65]. They showed that not only the micro/mesopores but also the macropores are filled with PANI. Tasdelen produced a cP(AAm-co-IA) by gamma radiation-induced polymerization [66]. Then, aniline was absorbed and polymerized by gamma ray irradiation. Without additional evidence, these authors assumed that an s-IPN was formed. On the other hand, Sharma et al. produced PANI inside a poly(acrylamide-aniline)-grafted gum ghatti hydrogel and observed the presence of nanospikes [67]. Xia et al. observed the formation of nanofibers during rapid ISP of aniline inside a PAA hydrogel [68]. Tang et al. produced a composite of PANI with a cPAAm matrix by ISP [69]. They observed, by XRD, the formation of crystalline domains of PANI, which should be formed in solid nanoparticles but not in s-IPN. 

A related synthesis method involves interfacial polymerization (ISP-I) [70], which involves either a liquid/solid (gel) interface [71] or a solid/solid interface between acrylamide and another gel (e.g., PVA). Since it is known that interfacial oxidative polymerization produces nanofibers [72], nanocomposites made of PANIs nanofibers dispersed in cPAM can be generated [73]. Miranda et al. electrospun core–shell fibers of PAAm-PAA in which the core contained aniline and the shell APS [74]. The formation of PANI occurred during electrospinning. Karbarz et al. polymerized aniline at the pore surface by loading APS in the hydrogel and filling the pores with a solution of aniline in nitrobenzene [75]. While cPAM hydrogels with macropores (“superporous” hydrogels) are usually produced by cryogelation, Mao et al. used a porous agarose hydrogel as sacrificial template to produce pores [76]. Then, ISP was used to incorporate PANI in the walls of the macropores, making the material electrically conductive.

− Hydrogel (cPAM) formation around nanoparticles of PANIs

**Scheme 2:**
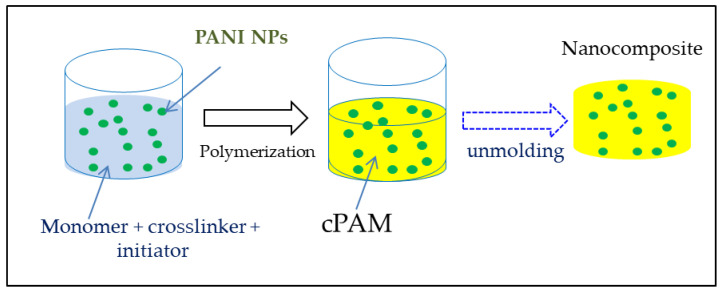
Mechanism of hydrogel (cPAM) formation around nanoparticles of PANIs.

Since formation of the hydrogel by radical polymerization is a well-established procedure, it seems possible to generate the hydrogel in the presence of PANI nanoparticles (Scheme 2). Experimentally, two problems have been observed. First, the amount of radical initiator has to be increased to obtain a self-standing hydrogel. This result suggests that quinonimine units in the PANI chains quench the growing chains. Moreover, the growing hydrogel absorbs water, thereby inducing the precipitation/aggregation of the PANI nanoparticles and generating an inhomogeneous material.

Abel et al. polymerized cPNIPAM around PANI NPs (dispersed by polymeric stabilization) [77].

**Scheme 3:**
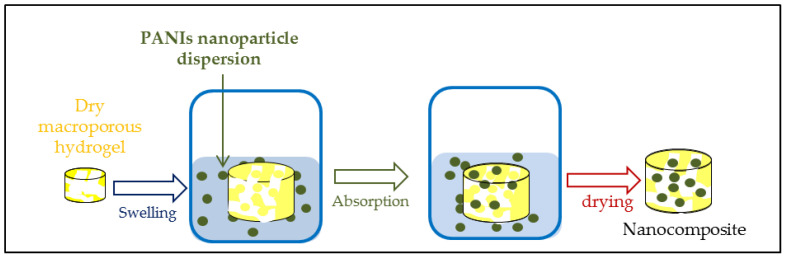
Mechanism of PANIs nanoparticle absorption into a macroporous hydrogel.

Absorption of PANIs nanoparticles inside cPAM hydrogels during swelling in nanoparticle dispersions is a simple way to produce nanocomposites [78] (Scheme 3). Typical cPAM hydrogels only permeate nanoparticles below 25–30 nm in diameter [79]. PANI nanoparticles produced by oxidative polymerization of aniline with polymeric stabilizers have diameters above 200 nm [80]. Indeed, PANI NPs (200 nm of diameter) only adsorb on the surface of nanoporous hydrogels [81]. On the other hand, superporous hydrogels made by cryogelation, easily absorb PANI NPs which become adsorbed on the macroporous surfaces [81].

#### 2.1.2. Formation of a Semi-Interpenetrated Network (s-IPN)

− Loading cPAM with PANIs from solution.

**Scheme 4:**
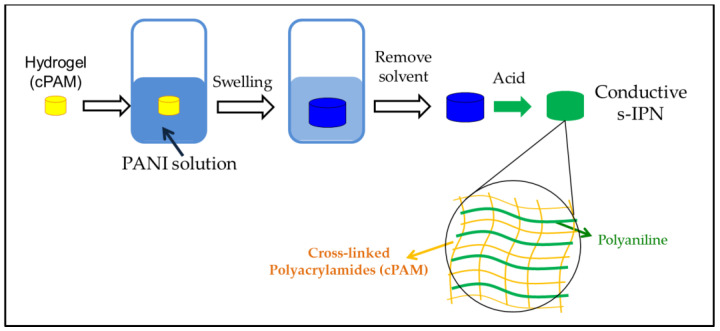
Mechanism of PANIs semi-interpenetration of PANIs inside a cross-linked polyacrylamides (cPAM) network, by swelling a dry hydrogel in a true solution of PANIs.

If a dry hydrogel network (cPAM) is swollen in a true solution of PANIs macromolecules, the linear conductive polymer becomes semi-interpenetrated in the network (s-IPN), as shown in Scheme 4.

One if the main features of hydrogels is their ability to swell in water (>10.000%). During swelling, small molecules and macromolecules can be incorporated from solution. However, PANI is not soluble in water. A functionalized polyaniline (sulfonated polyaniline, SPAN) [82], which is soluble in aqueous alkaline solution [82], was loaded into cross-linked polyacrylamide gels by swelling [83]. Since a linear soluble macromolecule was incorporated from the solution, the material is a true semi-interpenetrated network of SPAN in cPAAm. The swelling degree was affected by the presence of the SPAN chain, and the effect depended on the protonation state of the SPAN. At low pH, the imino groups of the polyaniline chain became protonated, and the conducting polymer became an inner salt. Therefore, no mobile counterions were required and a low internal osmotic pressure was apparent. On the other hand, at higher pH, the imino groups were deprotonated, and the sulfonate groups required mobile cations to balance the charge. The internal osmotic pressure resulted in the swelling degree at pH 12 being ca. 17 times greater than that observed at pH 1. However, at pH 12, SPAN was lost to the solution. It was found that cPNIPAM (but not cPAAm) swells strongly in NMP [84]. Since NMP is a good solvent of the deprotonated form of PANI (emeraldine base), PANI (EB) was interpenetrated into a cPNIPAM gel by swelling in a PANI solution [64]. Then, NMP was evaporated and the s-IPN swelled in water. Using an acidic solution, PANI became protonated and conductive. The SEM micrographs did not show separated domains as observed in the nanocomposite made by in situ polymerization. Moreover, the LCST of PNIPAM was affected by the interaction of PANI and PNIPAM chains, shifting from 32–33 °C for pure PNIPAM to 52–53 °C for the true s-IPN of PANI in cPNIPAM. The increase in LCST was likely due to the effect of hydrophilic moieties (amino and imino groups) on the hydrophobic/hydrophilic balance of the NIPAM monomer units. One way to make PANI soluble involves using amphiphilic anions (e.g., camphorsulfonate) as counterions [85,86]. Since PNIPAM (but not PAAm) can be swollen in CHCl_3_ [87], protonated (conductive) PANI can be incorporated into a cPNIPAM hydrogel.

#### 2.1.3. Grafted PANI Chains (gPC)

Another way to intimately mix cPAM and PANIs chains involves grafting PANI onto cPAM chains. Lu et al. copolymerized aniline with 2-aminophenylacryalamide using γ-radiation initiation. Then, aniline was polymerized by oxidative polymerization initiating from the aniline moieties pendant on the PAAm chains [88]. In a similar way, PNIPAM was radically polymerized in the presence of 4-aminothiophenol which acts as a chain transfer agent. The PNIPAM chains terminate in aniline moieties, and the oxidative polymerization of aniline started in the aniline end groups by producing block copolymers (PNIPAM-*b*-PANI) [89]. Smirnov et al. polymerized aniline by oxidation in the presence of linear soluble PAAm [90]. They reported NMR evidence of grafting of PANI chains onto the –NH_2_ groups of the acrylamide. Pang et al. synthesized PANI nanoparticles with vinyl groups at the end of the PANI chains [91]. Oxidative polymerization of aniline grew from these groups exposed at the PANI NPs surface, rendering a cross-linked hydrogel.

## 3. Technological Applications

The main goal of producing PANIs/cPAM materials involves using the properties, either of each of the materials, their combination or some synergic effect, for technological applications. In fact, most of the publications about these materials include applications based on the existing or emerging properties. While the properties of cPAM and PANIs enable the fabrication of various devices, it seems that some applications are more relevant than others. Photothermal actuators are based on the combination of the electromagnetic radiation (e.g., light) absorption and hydrogel thermosensitivity. Typically, PANI is combined with PNIPAM. Sensors can be built using hydrogels and PANIs. The simplest method involves dispersing the conducting polymer inside the porous hydrogel. The hydrogel, unlike PANIs, is elastic and deforms under compression or tension. Due to various mechanisms, the conductivity of the dispersed PANIs changes. Therefore, stretching, pressure or even body movements can be detected. The PANIs are conductive and electroactive in aqueous media. The dispersed PANIs networks inside the hydrogels could be used as electrochemical sensors of solution properties (e.g., pH) and/or as a way to wire redox enzymes which are trapped inside the hydrogel matrix. Moreover, both double-layer charging and oxidation/reduction of the conductive PANIs allows the storage of electrical charge in the material. In this way, supercapacitor devices can be built. While PANIs/cPAM materials have been also used for electrochemical solar cells, the amount of work investigating this use is limited. Since it is known that PANIs conductivity is affected by the interaction with molecules or ions, allowing easy access of analytes to the conducting network by dispersion inside the gel enables the detection of the presence (and/or concentration) of analytes through conductivity changes. Charged hydrogels act as soft arrangements of ionic charges linked to the mobile polymer chains. When a potential is applied, the charges tend to migrate to the electrode with the opposite charge, deforming the gel (electrophoretic drag). In this way, “soft” electrical actuators can be easily built. Simple hydrogels are commercially used for controlled release of medicinal drugs. “Smart” hydrogels change their swelling upon external stimuli (pH, temperature, or ion strength). The photothermal triggering could also control the release but with other stimuli (light, microwaves, radiofrequency) due to the presence of the conducting PANIs. 

### 3.1. Photothermal Actuators

Photothermal actuators require a material which absorbs electromagnetic radiation (e.g., light) to heat the material and another component which produces an effect (e.g., mechanical) upon heating.

#### 3.1.1. Light

**Scheme 5:**
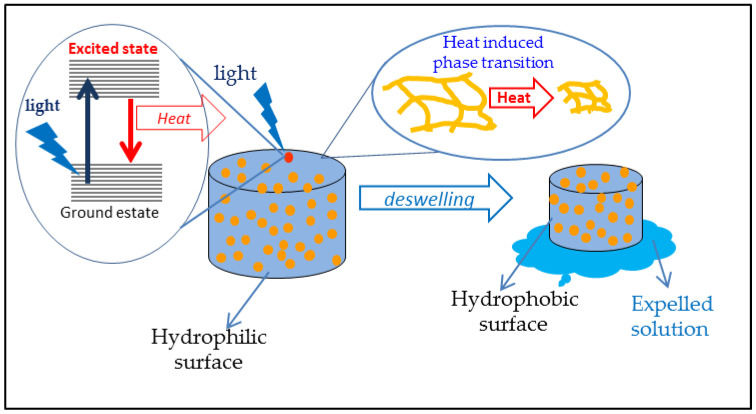
Mechanism of a photothermal actuator activated by light.

Polyanilines absorb light in the UV–visible and NIR range and heat up. Some cross-linked polyacrylamides (e.g., PNIPAM) suffer a transition (LCST) with a volume collapse, inner solution expulsions and change of hydrophobicity to a less hydrophilic surface. The combination of PANI and PNIPAM can be applied to produce a photothermal actuator (Scheme 5).

Zhao et al. produced a nanocomposite consisting of PNIPAM with PANI NPs loaded by ISP [92]. The hydrogel was produced either at room temperature (RT) or in a frozen solution by UV light-initiated radical polymerization. Then, aniline polymerizes by oxidation (APS) under RT or cryogelation conditions. Bilayer actuators were formed using a cross-linked (with GTA) PVA film as the inert layer. Illumination with a laser allows the observation of photothermally-induced movements. Deng et al. produced superporous cPNIPAM hydrogels made by cryogelation in frozen DMSO (mp = 19 °C) [93]. The gels were then loaded with PANI by ISP. The macroporosity increases the time required for swelling/deswelling to 2 min, and the presence of PANI gives the material photothermal sensitivity. Molina et al. fabricated PNIPAM nanoparticles cross-linked with dendritic polyglycerol (dPG) [94]. They then incorporated PANI by ISP inside the nanogels. The composite nanogels decrease in size both by heating the solution or illumination with a 758 nm (NIR) laser beam. The collapse of the cPNIPAM matrix occurs in both cases at 32–34 °C. The composite nanogels are not cytotoxic to human cells but show antitumor effects, both in vitro and in vivo, under illumination (T_max_ < 42 °C).

#### 3.1.2. Microwaves and Radiofrequency

**Scheme 6:**
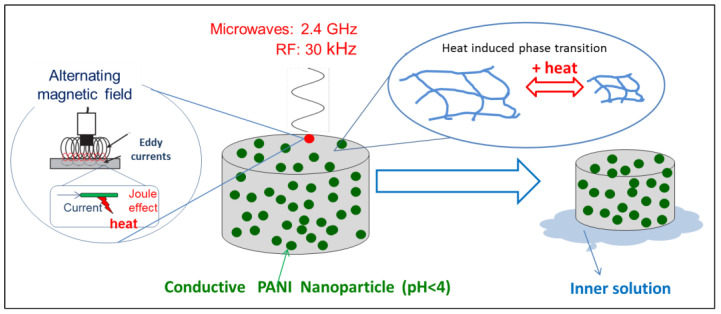
Mechanism of photothermal actuation using alternating magnetic fields.

Moreover, as electronic conductors, hydrogels generate currents (“Eddy”) when exposed to an oscillating electromagnetic field. The currents produce heat by the Joule effect. Therefore, electromagnetic radiation in both the microwave range (10 GHz to 500 MHz) and radio range (100 MHz to 10 kHz) produces heating. Upon exposure to electromagnetic radiation (microwaves, RF), the combined material heats up and the volume collapses, resulting in the expulsion of the inner solution and a change of the surface from hydrophilic to hydrophobic (Scheme 6).

Depa et al. prepared a cPNIPAM hydrogel by radical polymerization (RT and cryogelation) loaded with SiO_2_ NPs produced from silane precursors in situ [95]. They then adsorbed aniline onto the pore surface by swelling and deswelling in aniline solution. Using ISP, the pore surface was decorated with PANI. The amount of PANI seems low since the conductivity of the material was ca. 0.074 S cm^−1^. The same hydrogel loaded with PANI by complete swelling in aniline solution had a conductivity of 0.27 S cm^−1^. The nanocomposite is used to build an electrical switch which is triggered by external heat or by application of microwaves. Rivero et al. produced a cPNIPAM hydrogel by radical polymerization and transformed it into a nanocomposite of PANI by ISP [96]. The absorption of microwaves by PANI NPs heats up the NC and induces the LCST of cPNIPAM. Using this process, a microwave-driven electric switch is built. Nanocomposites produced by polymerization of cPNIPAM around PANI NPs [77] show sensitivity both to microwaves and RF irradiation. However, the NC collapse (cPNIPAM reaches LCST) occurred after 320 s with RF (320 kJ) but only required 30 s (21 kJ) when microwaves were applied. PANI can be incorporated by ISP into thin films of PNIPAM [52]. The absorption of light by PANI and heating (photothermal effect) makes it possible to structure the film topography using direct laser interference patterning (DLIP [97]). Moreover, the topography is different when PNIPAM is swollen (T < LCST) or collapsed (T > LCST) [20]. Upon irradiation with RF, the NC heats up above the LCST of cPNIPAM. Therefore, the topography of the film can be changed remotely by RF irradiation. 

### 3.2. Sensors

**Scheme 7:**
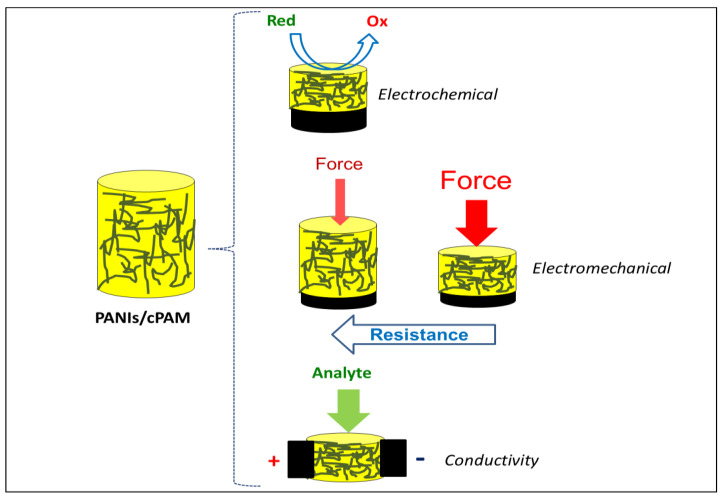
Different types of sensors based on PANIs/cPAM materials.

The PANIs/cPAM materials can also be used to build different kinds of sensors (Scheme 7):

(i) Electrochemical sensors: As can be seen, the conductive matrix can be connected to a base electrode. The large surface area of the conductive network can function as an electrode or the PANIs can be used to connect redox enzymes which are immobilized inside the cPAM matrix.
(ii) Electromechanical sensors: By compression of the combined material, the resistance decreases. In this way, pressure can be measured by monitoring the resistance.
(iii) Conductivity sensors: The extended PANIs network exposes a large surface to the hydrogel. Upon absorption of an analyte (volatiles, molecules, ions) into the gel matrix, the analyte interacts with the PANIs, changing its electronic properties (e.g., conductivity).

#### 3.2.1. Electrochemical

Since PANIs show electronic conductivity and redox activity, they can act as electrodes for direct (e.g., pH) or indirect (e.g., enzymatic) electrochemical sensing [98]. The dispersion of PANIs particles or macromolecules in the hydrogel allows the formation of tridimensional electrodes which show larger electrochemical areas and more rapid charge transport compared with PANIs thin films. 

Gniadek et al. prepared cPNIPAM hydrogels and absorbed metal (Au, Ag) salts into the matrix [99]. The pores were then filled with a solution of aniline in nitrobenzene. The metal salts are reduced to metal nanoparticles and PANI cover the surface of the pores. The LCST and mechanical properties of cPNIPAM remain unaltered. The conductive matrix, coupled with the presence of electrocatalytic metal NPs produces a sensor electrode for ethanol. The collapsed hydrogel (T > LCST) showed a higher electrocatalytic current. Das and Sarkar produced a cross-linked PVA hydrogel which is an IPN of PAAm on the surface of an electrode [100]. The aniline was then polymerized in situ by electrochemical oxidation. The material is used to immobilize the enzyme urease. Using DPV, it is possible to detect 1.5–1000 μM of urea with an LOD of 60 nM and a sensitivity of 878 μA mM^−1^ cm^−2^.

#### 3.2.2. Electromechanical

Since a hydrogel matrix is elastic, it can be compressed or stretched to a large degree (>100%). A PANI-containing material, consisting of nanoparticles in a nanocomposite or as macromolecules in an s-IPN, changes its conductivity with compression/stretching, leading to changes of the piece section (Ohm’s law) or formation/disruption of the percolation pathways. Therefore, the combined materials can be used as pressure, strain or motion sensors. 

Li et al. fabricated a nanocomposite by ISP of PANI into an acid-tolerant cellulose/PAAM hydrogel [101]. The electrical resistance of the PANI/cellulose/PAAm hydrogels depends on the compression/stretching. Therefore, such materials can be fabricated into motion sensors to monitor finger bending/pressing, fist clenching, throat swallowing/phonation in real-time. Chen et al. produced fibers of a ter-copolymer MEO_2_MA-OEGMA-NIPAM made by redox-initiated redox polymerization [102]. The linear polymer was transformed into fibers by stretching (1500%) in a Teflon tube. PANI was then formed inside the hydrogel fiber by ISP. The fibers show a good electrical conductivity (0.8799 S cm^−1^). The good mechanical properties (see below) and stretch-dependent conductivity enable application of the material in strain sensors. The electrical conductivity (measured by AC spectroscopy) of the nanocomposite PANI/PNIPAM [92] shows high sensitivity to elongation/compression allowing, the detection of body movements. Qin et al. fabricated a nanocomposite based on a cPAAm hydrogel mixed with bacterial cellulose and alginate [103]. The PANI nanoparticles were produced by ISP. The tensile strength was ca. 24 kPa, measured by DMA. The maximum gauge factor observed was ca. 0.8, while the response time was below 5 s, which demonstrates the possible application of the material as a strain sensor in different parts of the human body. Wang et al. produced a PNIPAM network by cross-linking with vinyl capped Pluronic F127 [104]. A PANI hydrogel, cross-linked by phytic acid, was interpenetrated in the hydrogel. The strain sensors showed good sensitivity to pressure (GF = 3.92), with response time of 0.4 s. The response remained stable for 350 cycles. Da Silva and Oréfice synthesized hydrogels composed of cP(AA-co-PNIPAM) and loaded them with PANI by ISP [105]. They assumed that an s-IPN was formed, but the data suggest that an NC was actually present. The material presented swelling due to temperature (related to the LCST of PNIPAM), pH changes (due to the protonation/deprotonation of PAA) and electric field effects(due to the presence of charges in PAA and PANI). Wang et al. prepared a cP(AAm-co-HEMA) network by radical polymerization and incorporated PANI by ISP [106]. The conductive hydrogels showed high sensitivity (GF = 11). Strain sensors based on the conductive hydrogels demonstrated reliable detection of human body movements, including joints, pulse and sound (voice) vibrations. A sensor array was fabricated to sense strain/pressures in two dimensions. The GF for different combined materials are summarized in Table 1.

#### 3.2.3. Conductivity

The conductivity of PANI is strongly affected by interaction with organic compounds [107]. PANI nanoparticles or macromolecules are more exposed to contaminants in the tridimensional structure of the hydrogel. Therefore, conductive materials consisting of a combination of PANIs with cPAM could be used as conductivity sensors. 

Demirci et al. synthesized a nanocomposite of rGO by radical polymerization of acrylamide in presence of a GO dispersion, followed by the in situ reduction to rGO [108]. The NC was loaded with PANI by ISP. The material was then used as a conductivity sensor to detect water-soluble herbicides and related chemical compounds. The conductivity of the P(AAm)-rGO/PANI NC decreased markedly (5.3-fold) upon interaction with a 50 ppm glyphosate solution.

### 3.3. Electrical Actuators

Hydrogels with fixed charges in the polymer chains suffer an electrophoretic drag when exposed to strong electric fields [109]. Since conducting polymers are doped by oxidation, they contain positive charges in the chains. Therefore, a neutral hydrogel combined with PANI will show mechanical changes when subjected to electric field, which enables the construction of electric actuators. On the other hand, conductive combined materials with a “smart” hydrogel matrix could act as switches activated by external stimuli (pH, temperature, ionic strength, etc.) that induce volume changes in the hydrogel [110,111,112]. Shi et al. built nanocomposites of PANI or PPy (with phytic acid as a dopant) with a PNIPAM/PAAm hydrogel matrix formed by ISP [113]. The material showed high conductivity (up to 0.15 S/cm for PANI) compared with similar combined materials. The use of phytic acid seems to produce a PANI hydrogel [114], which interpenetrates the cPAM hydrogel. The material has micrometric-sized pores, making the volume change during transition faster than in the case of nanoporous gels. Moreover, the conductive PANI inside the hydrogel increases the conductivity and improves the mechanical properties of the composite. An electrical switch can be built where heating the material induces the collapse of PNIPAM, breaking the electrical contact. Xu et al. produced fibers of nanocomposites by electrospinning [115]. Linear PNIPAM chains containing photo-crosslinkable units (4-acryloylbenzo-phenone) were elecrospun from solution in DMF (40%). The gel fibers were then cross-linked in mats using UV light. Finally, the fibers were swollen in aniline aqueous solution, and PANI was formed by ISP with APS. The electrical resistance depends on the PANI content and the swelling degree, which is controlled by the phase transition of PNIPAM. The relationship between conductivity and swelling is different for the contraction process (T > LCST) or expansion process (T < LCST). The materials can be used as electrical switches and also controlled resistors with memory effects. Zhu et al. loaded αCD into cPNIPAM by polymerization of NIPAM in the presence of αCD [116]. ISP of aniline was then used to produce a flexible (tensile strength 21.9 kPa) and conductive (d = 0.0064 S cm^−^^1^) nanocomposite. Surprisingly, the conductivity decreased upon volume collapse (>LCST of PNIPAM). The LCST of the nanocomposite is similar to that of PNIPAM, suggesting the weak interaction of PANI and PNIPAM chains, as expected when there is PANI NP dispersion. The thermosensitivity of PNIPAM enables the construction of a thermally-activated electric switch. Cut pieces of the nanocomposite could be healed by immersing in an acidic aniline solution, and a new PANI chain seem to form. A cPNIPAM hydrogel made by cryogelation in frozen DMSO by the authors in [93] showed pressure-dependent conductivity, and an electrical switch driven by the LCST of PNIPAM was demonstrated. A PNIPAM cross-linked by vinyl-capped Pluronic F127 [116] was used to build an electrical switch driven by the LCST of PNIPAM (“temperature alertor”). On the other hand, a simple nanocomposite made by ISP of aniline in a cPNIPAM hydrogel [96] showed a linear decrease in resistance with applied stress. Kim et al. produced a PANI/cP(AA-co-PVS) NC by ISP of aniline inside a hydrogel [117]. They assumed an IPN structure. The material showed clear and reversible changes in volume upon application of an electric field, suggesting an electrophoretic drag effect. Siddhanta et al. used ISP to incorporate PANI inside a cPAMPS hydrogel [118]. The hydrogel was prepared by photopolymerization. Most properties (e.g., swelling) of the composite remained similar to cPAMPS but electronic conductivity was added. Such behavior is consistent with a nanocomposite but not with an s-IPN, in which the close interaction between PANI and PAMPS chains should affect the properties. Application of 5 V/cm to the nanocomposite induced large water expulsion and volume contraction, and electric fields as low as 3 V/cm induced some volume contraction. 

### 3.4. Supercapacitors

PANIs could be used as electrode materials for supercapacitors due to their large theoretical specific capacitance [119]. However, thick PANIs pieces show slow charge transport (Do < 10^−^^10^ cm^2^/s). The combined PANI/cPAM electrodes expose a large surface area to the solution, due to the porosity of the gels, with a short (<200 nm) PANI path length ensuring fast responses. 

Zhang et al. [120] prepared a nanocomposite by in situ oxidative polymerization of aniline in a prestretched (500%) cPAAm hydrogel. After releasing the strain, the PANI NPs formed contacts between each other to form a continuous conductive network. Due to the good conductivity of the network, the material showed a high areal capacitance of 509.9 mF cm^−2^ (measured at 0.5 mA cm^−2^). Wang et al. prepared a conducting nanocomposite by ISP of aniline on a cPAAm gel which contained Ag NPs (stabilized with lignin) and HPA [121]. As in other cases, the effects of different components were proposed but remain unclear if all the components are required to obtain the composite properties. The hydrogel formation was made in a mixture of EG and H_2_O. The areal capacitance was 364.0 mFcm^−2^ (at 0.3 mA cm^−^^2^). The use of EG in the inner solution allowed it to operate as a supercapacitor below water freezing temperatures (down to −20 °C) The PANI/cellulose/PAAm materials produced by Li et al. [101] can be sandwiched between carbon cloth electrodes to fabricate supercapacitors and showed large areal (835 mF/cm^2^) and capacitance density (4.175 F/cm^3^). The areal energy of the composite was 74.22 μWh/cm^2^. The capacitance retention was above 96% after 5000 cycles. The supercapacitors could bear large bending/compressing deformations and operated normally from −60 to 80 °C. Hao et al. produced a nanocomposite (αCD-PAAm/PANI) to use as supercapacitor electrode [122]. A mixture of αCD with AAm and BIS was subjected to radical polymerization to form the hydrogel. Then, PANI was incorporated inside the hydrogel by ISP. The specific capacitance (Scap) was 315 F g^−^^1^ (measured by CV at 10 mV/s). Remarkably, the Scap only decreased to 245 F g^−^^1^ at 1 V/s, suggesting a fast counterion movement due to the open hydrogel structure. The material did not show significant degradation after 35,000 cycles. While the properties are suitable for commercial supercapacitors, it is unclear if the addition of the special αCD is the only way to produce such a material.

The capacitance of different materials is compared in Table 2.

The use of PANI-containing materials, including the combination of PANIs and cPAM in micro-supercapacitors, has been reviewed [123]. 

### 3.5. Bioactive Surfaces

The surfaces of cPAM are hydrophilic (due to the hydrogen bond interactions of water with the amide group) and neutral (unless a charged comonomer is used). The incorporation of PANIs decreases the hydrophilicity (due to the effect of the aromatic rings) and makes it weakly basic (due to the presence of diphenylamine units). Since the surface properties of the material modulate the biological response (adhesion, cytotoxicity, biofilm formation, etc.) of microorganisms, it is possible to produce bioactive surface by combining cPAM and PANIs.

Wu et al. produced a nanocomposite by ISP of aniline in an s-IPN of PAAm inside sulfonated chitosan [124]. The bactericidal activity of the PANI-containing NC against Gram-positive bacteria was demonstrated. Moreover, the conductive hydrogel was successfully used for in vivo electrical stimulation (ES) to promote infected chronic wound healing. The superporous cPNIPAM hydrogels made by cryogelation in frozen DMSO [93] did not negatively affect the cell adhesion of fibroblasts.

### 3.6. Drug Release

The controlled release of pharmaceuticals is the largest application of hydrogels [125]. The presence of PANIs inside hydrogels can change their hydrophobic/hydrophilic properties and/or to introduce positive charges which affect the partition equilibrium. Therefore, the release of active compounds (e.g., medicinal drugs) could be affected by the incorporation of PANIs into the cPAM gel matrix. 

Tang et al. produced a PANI/cPAAm NC by ISP of aniline inside a cPAAm hydrogel [126]. They investigated the release of a model dye (methylrosaniline chloride) from the NC. A non-Fickian behavior was observed. The rate of release depended inversely on the amount of PANI inside the hydrogel and directly on the temperature. Molina et al. produced nanocomposites of PANI and PNMANI loaded into cPNIPAM and cP(NIPAM-co-AMPS) hydrogels [127]. The partition equilibrium and release of dyes (Methyl Orange, Ruthenium-tris(2,2′-bipyridyl) ion, Dansyl Chloride) and pharmaceuticals (Tryptophan, Propranolol Chloride, Riboflavin) were measured. A clear effect of the charges in PANI and the relative hydrophobicity [128] of PNIPAM were detected. Lira et al. prepared a PANI/cPAAm composite by electrochemical oxidation of aniline inside a cPAAm hydrogel [129]. Raman spectroscopy allowed them to ascertain that the PANI grew from the electrode outwards, covering the surface of the pores, and not inside the polymer hydrogel matrix. They studied the release of a charged dye (safranine) and found that the diffusive release of the dye was strongly affected by the applied potential. 

### 3.7. Mechanical Reinforcement

The most common method of PANI loading into hydrogels is ISP of aniline inside the hydrogel. The method do not produce a s-IPN but becomes a nanocomposite with rigid PANI nanoparticles dispersed in an elastic hydrogel matrix. Therefore, the material becomes more elastic, though with an increase in the mechanical strength [130].

The fibers of the ter-copolymer MEO_2_MA-OEGMA-NIPAM, which are loaded with PANI by ISP [102], show a high fracture energy (172.43 kJ m^−^^2^) and good tensile strength (7.21 MPa). Liu et al. fabricated a cP(AAm-co-SMA) hydrogel loaded with carboxyl-functionalized multi-walled carbon nanotubes and solid PANI (dispersed by ultrasound) by entrapment during gel formation [131]. These authors claimed that the PANI was an s-IPN, which is, however, unlikely as PANI is not soluble in an aqueous solution. The P(AAm-co-SMA)/PANI/MWNTs-COOH composite hydrogel showed higher compressive strength (0.59 MPa) and larger strain (96.52%) than cP(AAm-co-SMA) unmodified hydrogels. Liu et al. also produced an NC of PANI and MWNT-COOH with a P(AAm-co-MAA) hydrogel matrix using the same procedure [132]. The compressive strength was higher (0.8 MPa). The nanocomposite made of cPAAm gel containing Ag NPs (stabilized with lignin) and HPA [121] had a tensile strength of 130.7 kPa and a fracture energy of 1862 J m^−^^2^, which is comparable to natural cartilages [133]. The compressive strength of a hydrogel (PNIPAM-co-2%AMPS) in water was ε = 6.5 kPa [64]. The incorporation of PANI nanoparticles by ISP of aniline inside the hydrogel increased the strength (ε = 11.3 kPa) [64]. However, the semi-interpenetration of rigid PANI chains in the same hydrogel, by absorption of PANI from its solution in NMP, increased the strength further (ε = 17.3 kPa) [64]. The nanoparticles act as rigid filler, lowering the fluidity of the elastic matrix [134]. However, rigid interpenetrated PANI chains change the mechanical properties of the polymer itself [135]. The mechanical strength (compressive or tensile) of different materials is compared in Table 3. As can be seen, the materials are soft (<1 GPa), except a gel also containing oligoethyleneoxide [102]. The incorporation of PANI always improves the mechanical properties (Table 3).

### 3.8. Electrochemical Solar Cells

Another electrochemical application where high surface area electrodes are relevant is electrochemical solar cells. The dispersion of PANI NPs in hydrogel-based nanocomposites is a way to produce large surface area electrodes using a simple process. Miranda et al. fabricated a material by UV-assisted polymerization of aniline inside a cP(AA-co-AAm) hydrogel [136]. The material showed electronic conductivity and a large electrochemical area (of PANI). Therefore, it is successfully used in a dye-sensitized solar cell (DSSC).

## 4. Conclusions and Future Outlook

The formation of composite materials by combining polyanilines (PANIs) and cross-linked polyacrylamides (cPAM) creates a synergy between the properties of both components. Using simple procedures, such as radical polymerization in the presence of a crosslinker (a divinyl compound) to produce cPAM and in situ polymerization (ISP) of anilines to incorporate PANIs, it is possible to produce a variety of composites. In the published work, it is often assumed that ISP of aniline loaded inside the cPAM produce s-IPN. However, there is much evidence that the product of ISP is a nanocomposite, with the hydrogel pores filled by PANI nanoparticles. A special case is the formation of PANI hydrogels, cross-linked by phytic acid, where two networks (cPANI and cPAM) interpenetrate each other. On the other hand, swelling cPAMs in a true solution of polyanilines (e.g., PANI (emeraldine base) in NMP) allows the formation of semi-interpenetrated networks (s-IPN). This approach is available for PANI since it has some solvents, whereas unmodified PPy or polythiophene are insoluble in most solvents. In these cases, ISP has to be used. In addition, other methods to produce nanocomposites have been tried, such as entrapment of PANI nano-objects during polymer gel formation or absorption of PANI nano-objects into macroporous gels. While a variety of cPAM have been used, >98% of the published work deals with polyaniline. Such an approach is reasonable since PANI has higher conductivity than functionalized polyanilines. 

Different forms of cPAM hydrogels have been modified by incorporation of PANI, including nanoporous and macroporous pieces, thin films, fibers and nanoparticles. While cPAM swell strongly in water, they can also swell in non-aqueous solvents. This property has been used to produce semi-interpenetrated networks of PANI in cPNIPAM by swelling the hydrogel in a solution of PANI(EB) in NMP or in a solution of PANI(ES) in CHCl_3_. Moreover, aniline polymerization inside cPAMs has been performed in ethylene glycol/water allowing the use of the composite as supercapacitor electrode at sub-freezing (−20 °C) temperatures. In recent years, PANI/cPAM composites have been combined with other nanomaterials, such as carbon nanotubes and graphene, or biopolymers, such as chitosan. While the combination of materials could improve the properties, sometimes it is unclear if all the components really contribute to the observed properties or only that novelty is sought. In each case, a detailed comparison of the simple composite and the one with the new component is required to assess its actual contribution. Photothermal actuators are one of the main applications of PANI/cPAM composites. Most of the excitation is performed with light (NIR to allow sufficient penetration in body tissue), but microwaves and radiofrequency have also been used. In non-biomedical applications, visible laser light could also be explored. Additionally, other thermosensitive cPAMs which have a UCST transition (e.g., poly(acrylamide-co-acrylonitrile) [137]) could be used to change the sign of the actuator movement or build tristate switches. The novel properties of the composites allow the fabrication of prototypes of different technological devices. The composites could also be used as electrodes for supercapacitors that show good capacitances (areal, specific, volumetric) with fast responses due to the large exposed area of PANI inside the hydrogel matrix. The incorporation of PANIs into the hydrogels has also been shown to affect the release of substances from the composite. 

There are three main drawbacks for the successful commercial application of devices based on PANI/cPAM composites. One, related to the hydrogel matrix, is their poor mechanical properties which even lead to disaggregation during swelling. However, the incorporation of PANI in the form of s-IPN networks or the formation of nanocomposites with large aspect ratio structures (e.g., nanofibers) improves the mechanical resistance. Moreover, the matrix could be constructed as a double network (DN), which shows exceptional mechanical properties [138]. Another drawback is related to the PANI dispersed component. There is a large loss of conductivity of PANI at neutral pH. Therefore, devices based on conductivity (even photothermal effects related to radiofrequency) would not work in biological media. To overcome the problem, PANI could be replaced by polypyrrole [81], which shows a smaller decrease (2–5-fold) of conductivity at neutral pH. Another possible approach is the use of self-doped polyanilines (e.g., sulfonated polyaniline [33]) which maintain conductivity in neutral pH [83]. A further impediment to commercial applications is the response time of the hydrogel, especially the recovery time to the swollen state. In most macroscopic systems (mm to cm sizes), this could take several hours. The slowness results from the slow mass transport of water inside a compact gel matrix and the slow relaxation of the polymer chains inside a compact gel. Deswelling is relatively fast since it occurs in a soft matrix filled with water, whereas swelling involves movements and mass transport in a compact gel. The best way to overcome the effect is reducing the path length for water diffusion. This can be achieved by using thin films [52], macroporous gels [81,93], or ensembles of gel nanoparticles [94].

## Figures and Tables

**Table 1:** Comparison of gauge factors measured by different conductive composites.

Material	Gauge Factor (GF)	Ref.
cPNIPAM-co-2%AMPS/PANI(NC)	5.64	[96]
cPNIPAM-co-2%AMPS/PANI(s-IPN)	0.95	[64]
PNIPAM c-Pluronic F127/PANI(NC)	3.92	[104]
cP(AAm-co-HEMA) (NC)	11	[106]

**Table 2:** Comparative performance of PANI/cPAM-based supercapacitors.

Material	Areal Capacitance F cm^−2^	Specific Capacitance (F g^−1^)	Ref.
Prestretched cPAAm/PANI	0.5099	--	[120]
cPAAM/HPA/AgNPs(lignin)/PANI	0.364	--	[121]
PANI/cellulose/PAAm/PANI	0.835	--	[101]
αCD-PAAm/PANI	--	315	[122]

**Table 3:** Effect of PANI on the mechanical properties of different combined materials.

Hydrogel Matrix	PANI Incorporation Method	Mechanical Strength (kPa)	Ref.
MEO_2_MA-OEGMA-NIPAM	ISP *	7210	[102]
cP(AAm-co-SMA)/MWNTs-COOH	Entrapment of solid PANI	590	[131]
cP(AAm-co-MAA)/MWNT-COOH	ISP *	800	[132]
cPNIPAM-co-2%AMPS	None	6.5	[64]
cPNIPAM-co-2%AMPS	ISP (NC)	11.3	[64]
cPNIPAM-co-2%AMPS	Absorption from solution (s-IPN)	17.3	[64]
cPAAm/HPA/AgNPs(lignin)	ISP *	130.7	[121]

* assumed to be an s-IPN by the authors but likely an NC.

## Abbreviations

AA	acrylic acid
AAm	Acrylamide
AC/DC	alternating current/direct current
AMPS	Acryamidopropanesulfonic acid
APS	ammonium persulfate
BIS	N,N’-Methylenebisacrylamide
cPAM	cross-linked polyacrylamides
CV	cyclic voltammetry
DMA	dynamic mechanical analysis
DMSO	dimethylsulfoxide
dPG	dendritic polyglycerol
DPV	differential pulse voltammetry
DSSC	dye-sensitized solar cell
EG	Ethylene glycol
GF	gauge factor
GO	Graphene oxide
HEMA	hydroxyethylmethacrylate
HPA	hydroxyapatite
IA	itaconic acid
IPN	interpenetrated network
ISP	in situ polymerization
LCST	lower critical solution temperature
LOD	lowest detection limit
MEO_2_MA	methoxyethoxy) ethyl methacrylate
MWNT-COOH	multiwall carbon nanotubes (carboxylated)
NC	nanocomposite
NIPAM	N-isopropylacrylamide
NIR	near infrared
NMP	N-methylpyrrolidone
NP	nanoparticles
OEGMA	oligo(ethylene glycol) methacrylate
PANI	Polyaniline
PANI(EB)	polyaniline in its deprotonated state (emeraldine base)
PANI(ES)	polyaniline in its protonated state (emeraldine salt)
PNMANI	poly(N-methylaniline)
PPy	polypyrrole
PTA	photothermal actuator
PVA	polyvinylalcohol
rGO	reduced graphene oxide
RT	room temperature
SEM	scanning electron microscopy
s-IPN	semi-interpenetrated network
SPAN	sulfonated polyanilline
TEMED	N, N, N’, N-tetramethylethylenediamine
UCST	upper critical solution temperature
UV	ultraviolet
αCD	α-cyclodextrin
